# Electrophysiology as a prognostic indicator of visual recovery in diabetic patients undergoing cataract surgery

**DOI:** 10.1007/s00417-021-05100-8

**Published:** 2021-04-06

**Authors:** Hao Wang, Fuliang Li, Jiawen Li, Jun Lin, Meifang Liu, Gang Wang, Min Wang, Li Ran, Anthony G. Robson, Shiying Li

**Affiliations:** 1 Southwest Hospital/Southwest Eye Hospital, Third Military Medical University (Army Military Medical University), Chongqing, China; 2 Key lab of Visual Damage and Regeneration & Restoration in Chongqing, Chongqing, China; 3 Department of Electrophysiology, Moorfields Eye Hospital, London, UK; 4 Institute of Ophthalmology, University College London, London, UK; 5 Present Address: Department of Ophthalmology, Xiang’an Hospital of Xiamen University, Medical Center of Xiamen University, School of Medicine, Xiamen University, Xiamen, China; 6 Present Address: Eye Institute of Xiamen University, Xiamen, China

**Keywords:** Visual electrophysiology, Cataract surgery, Diabetic patients, Prognostic indicator, Visual recovery

## Abstract

**Purpose:**

Visual outcomes after cataract surgery in diabetic patients with retinal or visual pathway disease are difficult to predict as the fundus may be obscured, and assessment of visual potential is challenging. This study assessed the value of visual electrophysiology as a prognostic indicator of visual recovery in diabetic patients with cataract, prior to cataract surgery.

**Methods:**

Forty-one diabetic patients (aged 52–80; 74 eyes) and 13 age-matched non-diabetic control patients (21 eyes) were examined prior to cataract surgery. Pre-surgical examinations included best-corrected visual acuity (BCVA), slit-lamp bio-microscopy, ISCEV-standard full-field electroretinography (ffERG), and flash visual evoked potential (flash VEP) testing. Electrophysiological assessments included quantification of the DA and LA ERG, oscillatory potentials (OPs; OP1, OP2, OP3, OP4) and flash VEP P1, P2, and P3 components. Post-operative BCVA was measured in all cases and the diabetic patients grouped according to the severity of visual acuity loss: mild (logMAR ≤ 0.1), moderate (0.1 < logMAR < 0.5), or severe (logMAR ≥ 0.5). A fourth group included those without diabetes. The pre-surgical electrophysiological data was compared between the four groups by analysis of variance.

**Results:**

The severity of post-surgical visual acuity loss in the diabetic patients was classified as mild (*N*=22 eyes), moderate (*N*=31 eyes), or severe (*N*=21 eyes). In the group without diabetes, post-surgical visual impairment was classified as mild (*N*=21 eyes). The pre-operative DA 10.0 ERG a-wave amplitudes, DA 3.0 ERG OP2 amplitudes, and the LA 3.0 a- and b-wave amplitudes showed best significant differences among the four groups. The flash VEP did not show significant difference between groups.

**Conclusion:**

Electrophysiological assessment of diabetic patients with cataract can provide a useful measure of retinal function. Full-field ERG components, including the DA 10.0 ERG a-wave, DA 3.0 ERG OP2 component, and the LA 3.0 a- and b-wave amplitudes, are of prognostic value in predicting post-surgical visual acuity, and may inform the surgical management of cataract patients with diabetes.

**Supplementary Information:**

The online version contains supplementary material available at 10.1007/s00417-021-05100-8.

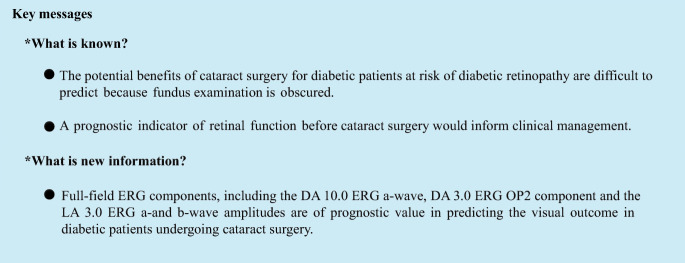

## Introduction

Type 2 diabetes is one of the most common diseases in older populations and is the major cause of blindness in young adults. Diabetic retinopathy (DR) and optic neuropathy are potentially irreversible sight-threatening complications, with an increased risk of cataracts. The potential benefits of cataract surgery are difficult to predict if fundus examination is obscured, and the management of such cases can be challenging [1, 2].

Full-field electroretinography (ffERG) and flash visual evoked potential (flash VEP) provide objective electrophysiological evaluations of retinal and optic nerve function, respectively [3, 4]. ffERG has been used in the assessment of retinal function associated with microvascular changes in type 2 diabetes [5] and has also been used in patients with cataracts [6, 7]. However, the value of the electrophysiological assessment of retinal and optic nerve function in diabetic patients with cataracts has yet to be established. Thus, the main aim of this study was to determine whether ffERG and flash VEP measures of retinal and optic nerve function are prognostic indicators of visual recovery in diabetic patients with significant lens opacities prior to cataract surgery.

## Methods

This study adhered to the Declaration of Helsinki principles. The Human Ethics Committees of Southwest Hospital approved the protocol, numbered KY2020054, and all subjects gave their written informed consent.

All subjects underwent routine clinical and pre-operative checks, including visual acuity testing, intraocular pressure assessment, slit-lamp examination, optometry, ophthalmology ultrasound B scans, intraocular lens-master (IOL-Master) examination, or ophthalmology ultrasound A scans to ensure the presence of moderate or severe cataracts and suitability for the study. Each subject also received blood pressure, electrocardiogram, and routine blood tests that included red and white blood cell and platelet, hemoglobin, blood glucose, glycosylated hemoglobin (HbA1c), and glycated albumin (GA) assessments.

The inclusion criteria included male or female patients with moderate or severe cataracts (C2N3P0-C3N3P3 in LOCS scoring) [8] deemed suitable for cataract surgery and aged between 50 and 80 years with type 2 diabetes and well-controlled blood glucose levels. The control group was age-matched non-diabetic patients with cataracts, and normal post-surgical best-corrected visual acuity (BCVA) (logMAR ≤ 0.1) was inclusion criteria for controls. The exclusion criteria included severe hypertension or other systemic diseases; a history of significant eye trauma, keratitis, high myopia, glaucoma, uveitis, retinal detachment, or other severe eye disease; and the inability to complete ophthalmic and electrophysiological examinations.

ffERG was performed in accordance with the International Society for Clinical Electrophysiology of Vision (ISCEV) 2015 standard [9], including dark-adapted (DA) 0.01 ERG, DA 3.0 ERG, DA 3.0 oscillatory potentials (OPs), DA 10.0 ERG, light-adapted (LA) 3.0 single flash ERG, and LA 30 Hz flicker ERG. The pupils were dilated to at least 7 mm in diameter in all cases. Oxybuprocaine hydrochloride eye drops (Santen Pharmaceutical Co., Ltd) were used as a topical anesthesia, and a jet electrode was used as the corneal electrode. Monocular flash VEP (Espion, Diagnosys, USA) recordings were made in accordance with the ISCEV 2016 standard [10] without mydriasis.

The pre-surgery examination, including ffERG and fVEP recording, and other examinations were performed one to 3 days before surgery, and the BCVA post-surgery values were recorded at 1 month ± 3 days following cataract surgery.

The diabetic patients were classified into one of three post-surgical groups on the basis of their BCVA values 1 month after cataract surgery: mild (LogMAR visual acuity, ≤ 0.1), moderate (0.1 < logMAR < 0.5), or severe (LogMAR visual acuity, ≥ 0.5). A fourth group included non-diabetic patients with cataracts. The amplitude and peak times of the main electrophysiological response components were measured, and the data was subdivided into the four post-surgical groups (Table 1) and compared by an analysis of variance (ANOVA).

**Table 1:** Pre-surgery clinical data for diabetic and control cataract subjects

Group*	No.	Eye	Age	F/M	VA pre	VA post	HbA1c (%)	GA (%)
Mild	15	22	67.7 ± 8.2	9/6	0.56±0.28	0.06±0.04	7.65 ± 1.75	17.18 ± 2.43
Moderate	20	31	65.9 ± 7.5	11/9	0.75±0.35	0.27±0.06	7.61 ± 1.48	17.58 ± 2.11
Severe	14	21	68.4 ± 7.8	7/5	1.43±0.42	1.12±0.48	7.88 ± 1.52	17.93 ± 2.85
Control	13	21	66.5 ± 6.9	7/6	0.62±0.19	0.05±0.04	4.92 ± 0.77	15.57 ± 2.02

*No.*, patient number in each group; *Eye*, eye number in each group; *VA pre*, average logMAR BCVA before cataract surgery; *VA post*, average logMAR BCVA after cataract surgery; *F/M*, female/male number of patients; *HbA1c*, glycosylated hemoglobin, which reflects the blood sugar change in the last 8–12 weeks prior to surgery; *GA*, glycated albumin, which reflects the blood sugar change in the last four weeks prior to surgery

^*^The grouping is based on the BCVA 1 month after cataract surgery: mild (LogMAR visual acuity, ≤ 0.1), moderate (0.1 < logMAR < 0.5), and severe (LogMAR visual acuity, ≥ 0.5)

All statistical analyses were performed using SPSS software (version 25.0; SPSS, Inc., Chicago, IL, USA). One-way ANOVA with Bonferroni correction was used to compare the electrophysiological parameters in the different groups. The post hoc testing was performed using the LSD method in SPSS, and statistical significance was set at *P* < 0.05. Post hoc testing was performed for conditions with significant effects (Bonferroni correction *P_adj* < 0.05) in the preceding ANOVA. Spearman’s rank correlation analysis was used to explore the correlation between the key electrophysiological parameters and the post-surgical logMAR BCVA values.

## Results

### Clinical details of diabetic and control subjects

A total of 41 diabetic patients (74 eyes) aged 52–80 years (median 69) and 13 control patients (21 eyes) aged 53–77 years (median 67) were recruited. The degree of lens opacity in all subjects was similar and classified as either a moderate or severe cataract (from C2N3P0-C3N3P1) [8]. Visual acuity prior to cataract surgery ranged between logMAR 2.0 and 0.3. The post-surgical classification of the diabetic patients based on the BCVA values 1 month after cataract surgery resulted in 15 patients (22 eyes) in the mild group (aged 53–79 years; median 70); 20 patients (31 eyes) in the moderate group (aged 52–77 years; median 66.5); and 14 patients (21 eyes) in the severe group (aged 53–80 years; medians 68.5). ANOVA was performed for the age of four groups (F = 0.659, *P* = 0.825). Post-surgical BCVA in the control group accorded with the inclusion criteria for the controls (logMAR ≤ 0.1). See Table 1. The clinical details on the typical subjects of the four groups are shown in Table S1.

### Comparison of full-field electroretinography waveforms

**Fig. 1:**
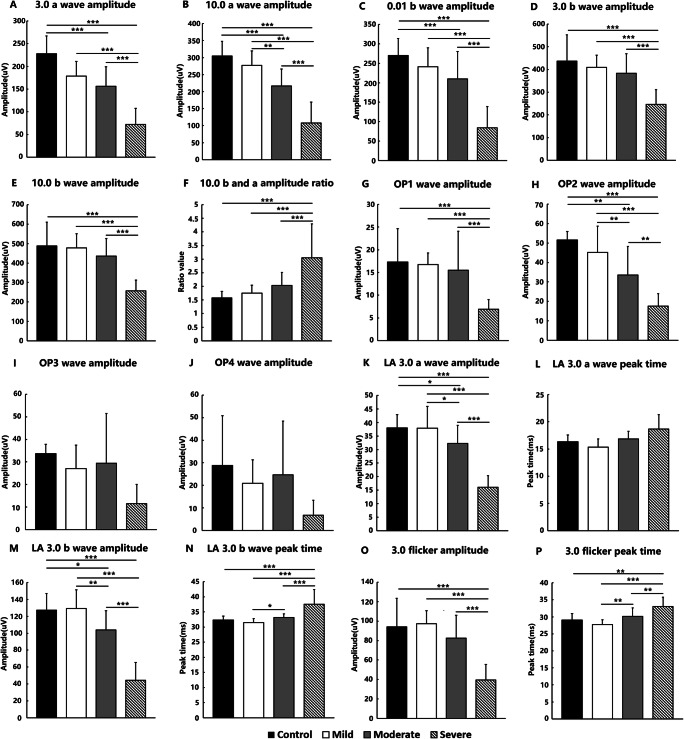
The mean dark-adapted (DA) and light-adapted (LA) electroretinography (ERG) component characteristics are shown for the control group and each of the three post-operative diabetic BCVA groups. **a** DA 3.0 a-wave amplitude. **b** DA 10.0 a-wave amplitude. **c** DA 0.01 b-wave amplitude. **d** DA 3.0 b-wave amplitude. **e** DA 10.0 b-wave amplitude. **f** DA 10.0 b and a wave amplitude ratio. **g** OP1 wave amplitude. **h** OP2 wave amplitude. **i** OP3 wave amplitude. **j** OP4 wave amplitude. **k** LA 3.0 a-wave amplitude. **l** LA 3.0 a-wave peak time. **m** LA 3.0 b-wave amplitude. **n** LA 3.0 b-wave peak time. **o** LA 3.0 30Hz flicker amplitude; LA 3.0 30Hz flicker peak time. The statistically significant differences between the groups are indicated as *P_adj < 0.05, **P_adj < 0.01, and ***P_adj < 0.001

Figure 1 shows the pre-operative DA 0.01, 3.0, and 10.0 a- and b-wave amplitudes, the OP 1–4 wave amplitude, and the LA 3.0 a- and b-wave amplitudes along with the LA 30Hz flicker amplitude and peak times for all groups. Significant differences are highlighted by asterisks and are summarized in Table 2. The representative electrophysiological results of the four groups are shown in Supplementary Figure S1.

**Table 2:** Summary of ANOVA F value and *P_adj* value in ffERG

Parameter	F value	*P_adj* value
0.01 b am	11.816	< 0.001
3.0 a am	17.497	< 0.001
3.0 b am	7.200	0.006
10.0 a am	23.989	< 0.001
10.0 b am	10.104	0.003
10.0 b/a	10.106	< 0.001
OP1	3.353	0.026
OP2	9.849	< 0.001
OP3	2.219	1
OP4	1.684	1
LA 3.0 a am	17.648	< 0.001
LA 3.0 a pe	5.683	0.32
LA 3.0 b am	19.609	< 0.001
LA 3.0 b pe	12.293	< 0.001
Flicker am	10.495	< 0.001
Flicker pe	7.693	0.003

In the DA 0.01 condition, the b-wave amplitude ANOVA was performed with Bonferroni correction (F = 11.816, *P_adj* < 0.001). The differences in the b-wave amplitudes between the mild and severe groups and between the moderate and severe groups were significant. The difference between the mild and moderate groups was not significant (Fig. 1c). In the DA 3.0 condition, ANOVA was performed with Bonferroni correction for the a-wave (F = 17.497, *P_adj* < 0.001) and b-wave (F = 7.200, *P_adj* = 0.006) amplitudes. Both the a- and b-wave amplitudes were significantly different between the mild and severe groups and between the moderate and severe groups. However, the difference between the mild and moderate groups was not significant (Fig. 1a, d). In addition, the difference between the control and mild groups was not significant. For the DA 10.0 ERG b-wave amplitude, ANOVA was performed with Bonferroni correction (F = 10.104, *P_adj* = 0.003), and there were significant differences between the mild and severe groups and between the moderate and severe groups but not between the mild and moderate groups (Fig. 1e). The DA 10.0 ERG a-wave amplitude ANOVA was performed with Bonferroni correction (F = 23.989, *P_adj* < 0.001), and significant differences between the mild, moderate, and severe groups were found (Fig. 1b). Thus, all three groups could be distinguished by the DA 10.0 a-wave amplitude. The DA 10.0 b-wave to a-wave amplitude ratio was also calculated (Fig. 1f), and ANOVA was performed with Bonferroni correction (F = 10.106, *P_adj* < 0.001). There was no significant difference between the mild and moderate groups, but there were significant differences between the mild and severe groups and between the moderate and severe groups. Peak time analysis was also performed for the DA response components, but there were no significant differences between the groups.

The amplitudes of the DA 3.0 OP1, OP2, OP3, and OP4 components in the four patient groups were compared. In terms of the DA 3.0 OP1 (F = 3.353, *P_adj* = 0.026, ANOVA, Bonferroni correction), significant differences were found between the mild and moderate groups and between the moderate and severe groups (Fig. 1g). In terms of the OP3 (F = 2.219, *P_adj* = 1, ANOVA) and OP4 (F = 1.684, *P_adj* = 1, ANOVA) amplitudes, no significant differences were found (Fig. 1i, j). Regarding the OP2 wave amplitude (F = 9.849, *P_adj* < 0.001, ANOVA), there were significant differences between the mild, moderate, and severe groups (Fig. 1h). The OP2 was the most consistent OP wave; therefore, it was considered a key parameter in the pre-cataract surgery assessment of retinal function.

For the LA 3.0 ERG, an a-wave amplitude ANOVA was performed with a Bonferroni correction (F = 17.648, *P_adj* < 0.001), and a b-wave amplitude ANOVA was also performed with Bonferroni correction (F = 19.609, *P_adj* < 0.001). The amplitudes of both the a- and b-waves showed significant differences between most groups, with the exception of between the mild and control groups (Fig. 1k, m). Regarding the LA3.0 ERG a-wave peak time (F = 5.683, *P_adj* = 0.32), no significant differences were found (Fig. 1l); for the b-wave peak time (F = 12.293, *P_adj* < 0.001), significant differences were found in the mild, moderate, and severe groups, but no significant differences were found between the control and moderate groups or between the control and mild groups (Fig. 1n). In the comparisons of the LA 3.0 30Hz flicker, an amplitude ANOVA was performed with Bonferroni correction (F = 10.495, *P_adj* < 0.001), a peak time ANOVA also was performed with Bonferroni correction (F = 7.693, *P_adj* = 0.003). Amplitude revealed significant differences between the control, mild, moderate, and severe group, respectively, but not a significant difference among the control, mild, and moderate group (Fig. 1o). Significant differences in the peak time between the mild and moderate group and between the moderate and severe group were observed, but there was no significant difference between the control and moderate group (Fig. 1p). Table 2 summarizes the ANOVA F value and adjusted *P* value, and Table 3 summarizes the significance of the ffERG component differences between the different groups.

**Table 3:** Summary of *P_adj* values for the two-group comparisons in ffERG. Mild (*Mi*), moderate (*Mo*), severe (*Se*), and control (*Co*)

Parameter	Mi–Mo	Mo–Se	Mi–Se	Co–Mi	Co–Mo	Co–Se	Comparison
0.01 b am	/	***	***	/	***	***	√
3.0 a am	/	***	***	/	***	***	√
3.0 b am	/	***	***	/	/	***	√
10.0 a am	**	***	***	/	***	***	√√
10.0 b am	/	***	***	/	/	***	√
10.0 b/a	/	***	***	/	/	***	√
OP1	/	***	***	/	/	***	√
OP2	**	**	***	/	**	***	√√
OP3	/	/	/	/	/	/	×
OP4	/	/	/	/	/	/	×
LA 3.0 a am	*	***	***	/	*	***	√√
LA 3.0 a pe	/	/	/	/	/	/	×
LA 3.0 b am	**	***	***	/	*	***	√√
LA 3.0 b pe	*	***	***	/	/	***	√
Flicker am	/	***	***	/	/	***	√
Flicker pe	**	**	***	/	/	**	√

*/*, *P_adj* > 0.05; *, *P_adj* < 0.05; **, *P_adj* < 0.01; ***, *P_adj* < 0.001; *×*, no significant statistical difference among the groups; *√*, significantly statistical difference between “severe” and any another group; *√√*, significantly statistical difference between every group except “Co-Mi”; *am*, amplitude; *b/a*, b- and a-wave amplitude ratio; *LA*, light-adapted; *pe*, peak time

*am*, amplitude; *b/a*, b- and a-wave amplitude ratio; *LA*, light-adapted; *pe*, peak time

The summary of the *P_adj* values for the four groups shows the *P_adj* values for each two-group comparison. A greater difference in the *P* values represents the higher validity of the parameters. The valid parameters included the 0.01 b-wave amplitude, 3.0 a- and b-wave amplitude, 10.0 b-wave amplitude, 10.0 b- and a-wave amplitude ratio, OP1 wave amplitude, LA b-wave peak time, and LA 3.0 flicker amplitude and peak time. The most valid parameters were the DA 10.0 a-wave amplitude, OP2 wave amplitude, and LA 3.0 a- and b-wave amplitudes. In terms of these four parameters only, each two-group comparison except that of the control and mild groups indicated significant differences. The lack of a significant difference between the control and mild group was a reasonable outcome. The comparisons in terms of the four valid parameters indicated that the significant differences were higher for the 10.0 a-wave due to the greater difference in the ***P_adj values.

**Fig. 2:**
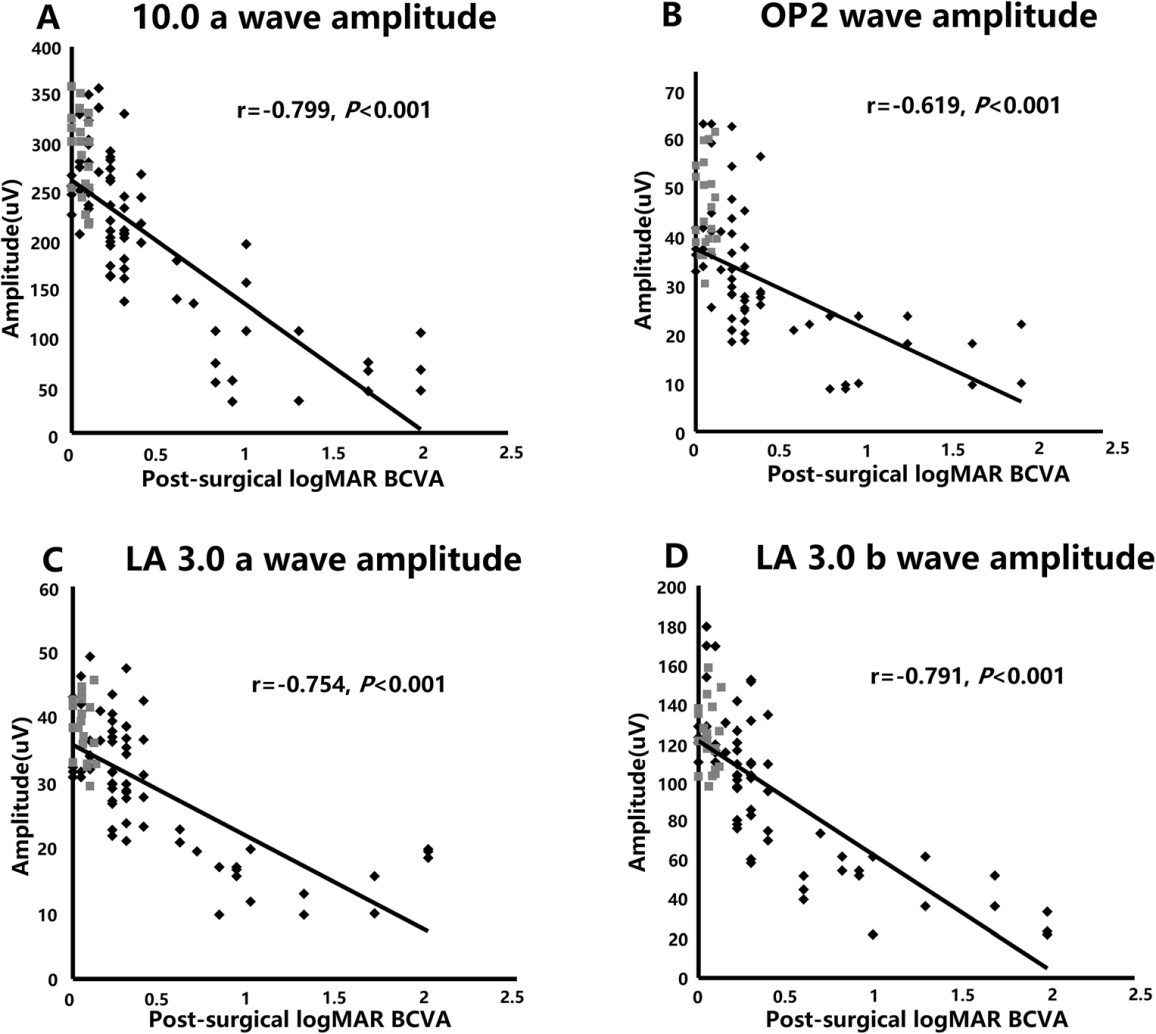
Plots showing the negative correlation for all the subjects between logMAR BCVA and each of the four key ERG parameters, including the DA 10.0 a-wave amplitude (**a**), OP2 amplitude (**b**), LA 3.0 a-wave (**c**), and b-wave (**d**) amplitudes. Black dots, diabetic patients; gray dots, controls

The most consistent predictions of post-surgical BCVA were based on the DA 10.0 a-wave amplitude, OP2 wave amplitude, and LA 3.0 a- and b-wave amplitudes (Table 3). For all the subjects, including controls and diabetic patients, Spearman’s rank correlations between these four key parameters and the post-surgical visual outcomes were examined (Fig. 2). The logMAR BCVA showed significant negative correlations with the amplitudes of the DA 10.0 ERG a-wave (*r* =−0.799, *P* < 0.001; Fig. 2a), OP2 (*r* =−0.619, *P* < 0.001; Fig. 2b), and LA 3.0 a-wave (*r* =−0.754, *P* < 0.001; Fig. 2c) and b-wave (*r* =−0.791, *P* < 0.001; Fig. 2d).

### Comparisons of P1, P2, and P3 waves in terms of flash cortical visual evoked potential

**Fig. 3:**
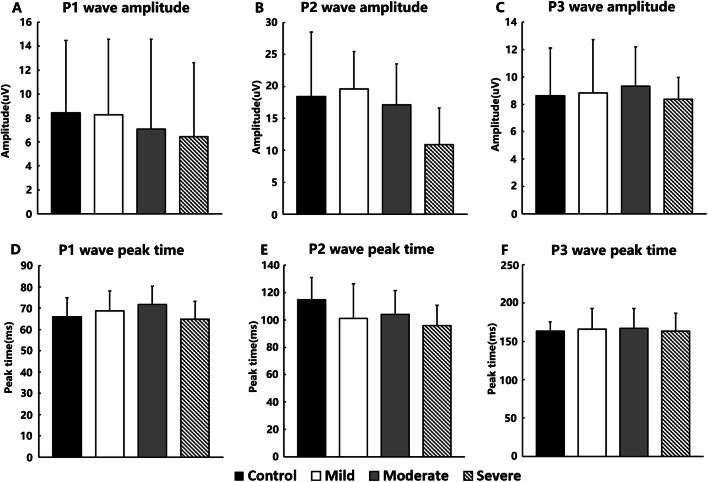
P1, P2, and P3 wave amplitude and peak times in terms of the flash cortical visual evoked potential of the control, mild, moderate, and severe groups. **a** P1 wave amplitude. **b** P2 wave amplitude. **c** P3 wave amplitude. **d** P1 wave peak time. **e** P2 wave peak time. **f** P3 wave peak time

The amplitude and peak times of the P1, P2, and P3 waves in terms of the flash VEP of the mild, moderate, severe, and control groups were compared via ANOVA (Fig. 3).

No significant difference in amplitude was found in the flash VEP P1 (F = 0.162, *P* = 0.921), P2 (F = 2.139, *P* = 0.086), and P3 (F = 0.928, *P* = 0.887) components between the four groups (Fig. 3a, b, c). No significant difference was found in the peak times of the flash VEP P1 (F = 1.824, *P* = 0.154), P2 (F = 2.389, *P* = 0.079), and P3 (F = 0.675, *P* = 0.847) components between the four groups (Fig. 3d, e, f).

## Discussion

This study examined the value of standardized electrophysiological techniques in the assessment of retinal function in diabetic patients with cataracts. The detailed quantification of full-field ERG parameters enabled the objective assessment of retinal dysfunction and the identification of several ERG components of prognostic value in predicting post-surgical visual outcomes.

Several DA and LA ffERG components were found to be prognostic in predicting BCVA following cataract surgery, and a similar utility of electrophysiology has been reported in evaluations of non-diabetic patients pre- and post-cataract surgery [11, 12]. It is notable that the significant differences between the outcome groups in the current study were greatest in terms of the dark-adapted strong flash (DA 10.0) ERG a-wave. The value of the DA 10.0 ERG a-wave in this context may relate to the retinal response approaching a maximum amplitude irrespective of the media opacity, whereas less-saturated responses to dimmer flashes could be more affected by a reduction in light transmission. The DA 10.0 ERG a-wave normally receives a far greater contribution from the rods than the cones. Although BCVA is normally cone-mediated, the rod-dominated DA 10.0 ERG a-wave may have given the most sensitive indication of underlying diffuse retinal dysfunction and retinopathy in these study patients, which was related to their visual outcomes following surgery. In moderate and severe cases, there may be macular oedema or macular lesions [13, 14], which are likely to influence BCVA irrespective of the ERG findings. The inclusion of a few such macular oedema or macular lesions cases highlights the prognostic value of the ERG, which shows strong correlations despite this potentially confounding variable. The mfERG has been used to assess macular and posterior pole cone system function pre-cataract surgery, but such recordings are likely to be influenced by cataract [12, 15]. A hand-held ffERG device was used to assess diabetic retinopathy, and significant correlations were found between the DR and ffERG waves [16, 17]. In this study, we also found significant correlations between the DR and ffERG waves in the cataract patients, with the DA 10.0 as a new-found more effective mode than other modes in cataract patients.

Optic neuropathy is a potentially irreversible sight-threatening complication of diabetes, but routine assessment may also be confounded by the presence of cataracts. Flash VEP are generally less sensitive than pattern VEP to optic nerve dysfunction and are far less sensitive to the effects of optical degradation [18]. Significant cataract precludes the reliable pre-surgical use of a pattern VEP, but the presence of normal flash VEP peak times and amplitudes in the current series helped to exclude marked optic nerve dysfunction as an exacerbating cause of vision loss.

In spite of the limited number of participants in this study, highly significant differences were observed. A limitation of this study is that the post-surgery retinal structure examinations were performed for each patient; however, the photographic data was incomplete. However, the representative eye structure examination results are shown in Supplementary Figure S2. The evaluation of some of the patient examinations indicated a relationship between the diabetic retinopathy severity and pre-surgery ffERG and post-surgery visual acuity results. Thus, the post-surgery retinal structure parameters might be used to analyze the correlations between the pre-surgery ffERG parameters in the future.

## Conclusion

This study shows that pre-surgical, international-standard ffERGs are of value in the management of patients with diabetic retinopathy and cataracts. Multiple ffERG components are related to post-surgical visual outcomes and highlight a relationship between the severity of retinal dysfunction and visual prognosis, which is consistently revealed by the dark-adapted strong flash (DA 10.0) ERG a-wave amplitude, OP2 component, and LA 3.0 a- and b-wave amplitudes. Normal pre-operative flash VEP help exclude marked post-retinal dysfunction as a cause of visual pathology and highlight the value of comprehensive electrodiagnostic testing.

## Supplementary Information

ESM 1 (DOCX 3102 kb)
