# Supplementary material for: Electrophysiology as a prognostic indicator of visual recovery in diabetic patients undergoing cataract surgery
Source: Graefes Arch Clin Exp Ophthalmol. 2021 Apr 6;259(7):1879–87. doi: 10.1007/s00417-021-05100-8 (PMC8277643; doi:10.1007/s00417-021-05100-8)
Supplement: Supplementary file 1 — (DOCX 3102 kb) [file 417_2021_5100_MOESM1_ESM.docx]

1. clinical details of the typical subjects of the four groups

Table S1 Clinical details of the typical subjects of the four groups

| Group* | Age | Gender | Eye | Cataract  degree | LogMAR BCVA  (pre-surgery) | LogMAR BCVA  (post-surgery) | HbAlc (%) |
| --- | --- | --- | --- | --- | --- | --- | --- |
| Mild vision  impaired | 62 | female | left | C2N3P1 | 0.5 | 0.1 | 7.75 |
| Moderate vision impaired | 63 | male | left | C2N3P0 | 0.8 | 0.3 | 7.98 |
| Severe vision impaired | 66 | female | left | C2N3P0 | 1.2 | 0.7 | 8.01 |
| Control | 65 | male | left | C2N3P1 | 0.5 | 0 | 4.55 |

We chose one subject from each group to show the clinical details in table S1. We can find the age and cataract severity of the subjects is similar. In one-month post-surgery, the LogMAR BCVA is dramatically different, so the patients were grouped to different groups.

2. Typical full-field electroretinography and flash cortical visual evoked potential waveforms

Supplementary figure S1 shows representatively examples of ISCEV standard ERG and flash VEP waveforms from each of the four patient groups representative examples of from.


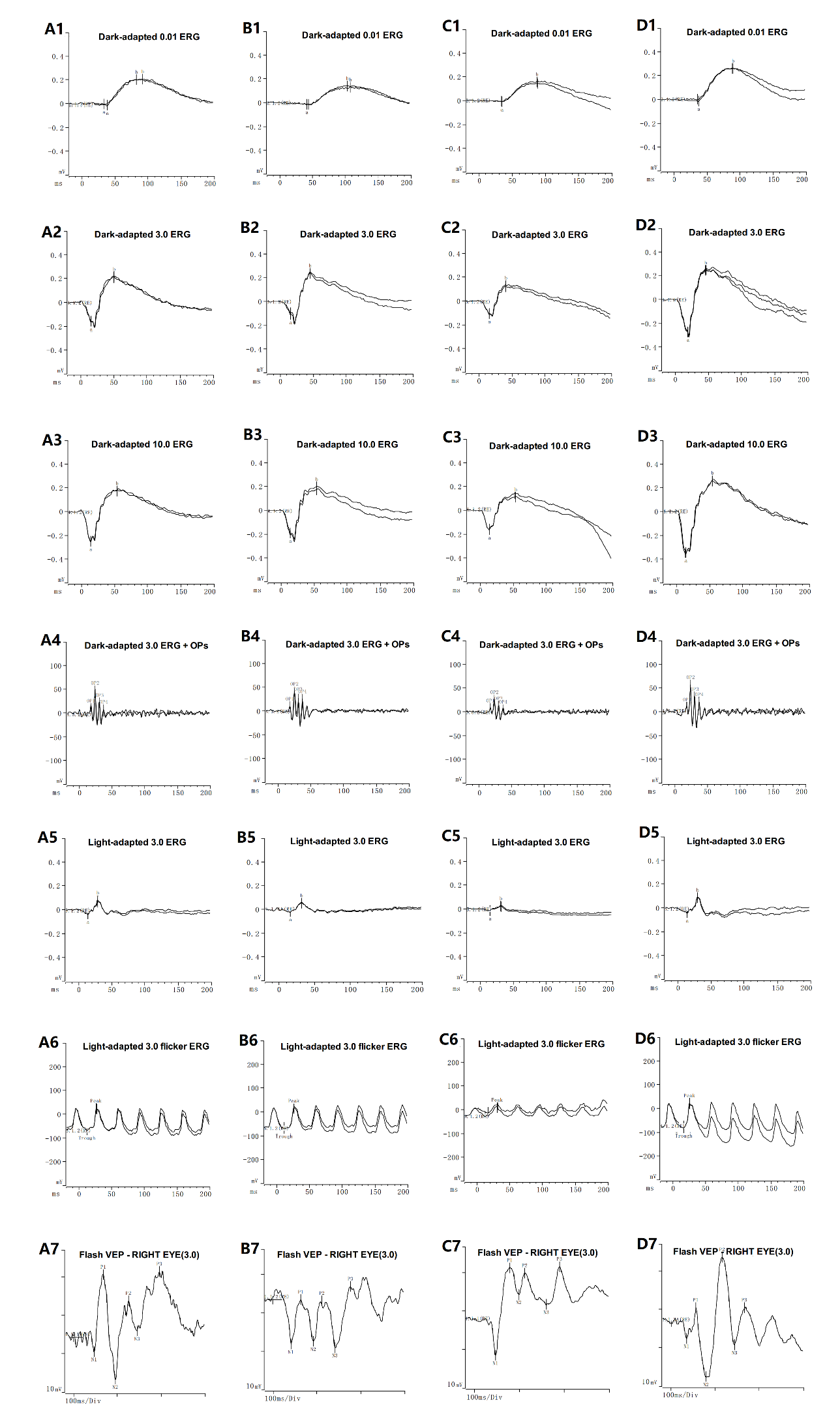


**Figure S1. Typical full-field electroretinography (ffERG) and flash visual evoked potential (flash VEP) waveforms obtained prior to cataract surgery, from each of the four patient groups: A1–A7, mild visual dysfunction group; B1–B7, moderate visual dysfunction group; C1–C7, severe visual dysfunction group; and D1–D7, control group. All waveforms are superimposed to demonstrate reproducibility.**

As is shown in Figure S1, the ffERG waveforms for the four groups were identified. The amplitude of the waves in the mild group (Fig. S1 A1–A6) seemed to be larger than that in the severe group (Fig. S1 C1–C6) but similar to that in the control group (Fig. S1 D1–D6). The amplitude of the a-wave in the 10.0 condition and that of the OP2 wave are remarkable. The amplitude of the waves in the moderate group (Fig. S1 B1–B6) seemed to be greater than that in the severe group. However, it was slightly lower than the amplitude in the mild and control groups. The differences in the peak times were not as obvious as those in amplitude.

The flash VEP waves were not as stable as the ffERG waves. There were variations in the P1, P2 and P3 amplitude in the four groups (Fig. S1 A7, B7, C7, D7). There was no obvious difference in the P1 and P3 amplitude. However, the P2 wave amplitude in the severe group (Fig. S1 C7) was slightly lower than that in the other groups. The peak time differences in the flash VEP were also unremarkable.

3. Images of the typical pre- and post-cataract surgery eye structures

Representative pre-cataract surgery photographs of the anterior segment, macular optical coherence tomography (OCT) and B-scan ultrasonography of the mild, moderate, severe and control groups are presented in supplementary figure S2. The post-surgery colour fundus photographs (CFPs) are shown in figure S2. Images from the high-definition macular OCT and fundus fluorescein angiography (FFA) of the retina are also included.


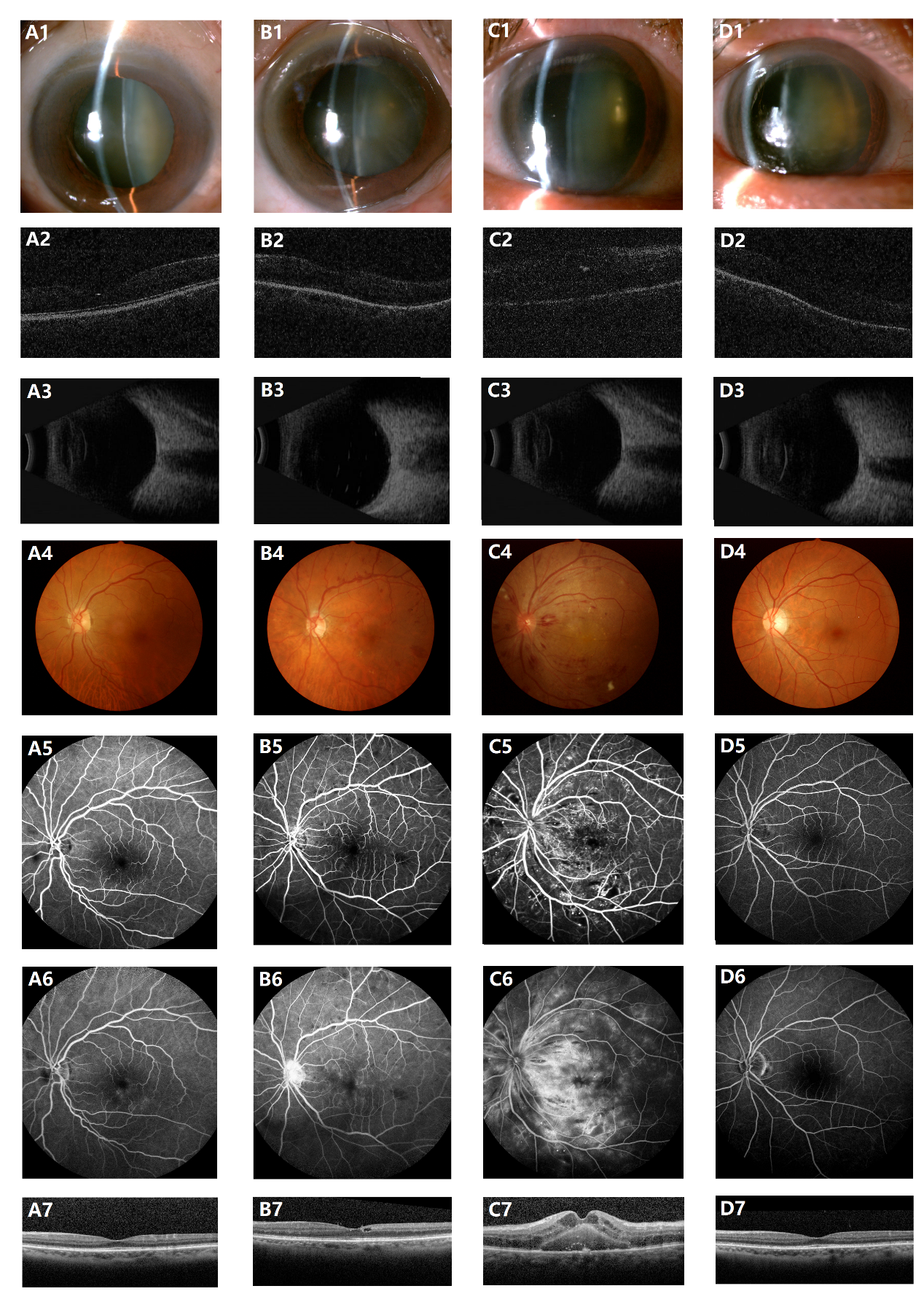


**Figure S2. Examples of pre- and post-cataract surgery multimodal imaging in the four groups: A1–A7, mild; B1–B7, moderate; C1–C7, severe; and D1–D7, control. Rows 1 to 3 are the pre-operative images: Row 1, anterior segment of the eye; Row 2, macular optical coherence tomography (OCT); and Row 3, B-scan ultrasonography of the globe. Rows 4 to 7 show the post-surgery images of the same subjects: Row 4, colour fundus; Row 5, early stage of fundus fluorescein angiography (FFA); Row 6, later stage of FFA; and Row 7, macular OCT.**

As shown in Figure S2, cataract severity was found to be similar in the patients: C2N3P0 to C3N3P1 (Fig. S2 A1, B1, C1, D1). The quality of the pre-surgery macular OCT was suboptimal because of lens opacity (Fig. S2 A2, B2, C2, D2). The B-scan ultrasonography indicated the absence of vitreous haemorrhage or retinal detachment (Fig. S2 A3, B3, C3, D3).

The post-surgery CFP suggested that the visible retinopathy and severity of the fundus abnormalities were related to the post-operative visual acuity (Fig. S2 A4, B4, C4, D4). The early-stage FFA (Fig. S2 A5, B5, C5, D5) and later-stage FFA (Fig. S2 A6, B6, C6, D6) indicated that there was a relationship between the degree of diabetic retinopathy and the level of visual dysfunction. Diabetic retinopathy was the most serious in the severe group and slight in the mild group. The post-surgery high-definition macular OCT revealed a relationship between macular oedema progression and visual dysfunction (Fig. S2 A7, B7, C7, D7). There was obvious macular oedema in the mild group; however, it was very serious in the severe group.
